# Supplementary material for: Long-read viral metagenomics captures abundant and microdiverse viral populations and their niche-defining genomic islands
Source: PeerJ. 2019 Apr 25;7:e6800. doi: 10.7717/peerj.6800 (PMC6487183; doi:10.7717/peerj.6800)
Supplement: Supplementary Methods — Describes the quality control of sequence data and methods used to evaluate (1) relative abundances of contigs; (2) whether long read assembly captured more microdiverse genomes; (3) recovery of genomic islands and their predicted functional composition at the population level; (4) evaluation of functional variance of viral genomic islands spanned by VirION reads. [file peerj-07-6800-s001.pdf]

Supplementary Methods

Quality control of sequencing data

Illumina sequencing data was processed with Trimmomatic (Bolger, Lohse & Usadel, 2014) to remove adapter sequences and low-quality reads. Reads for assembly were normalised to ~100-fold coverage and error corrected with bbnorm (https://github.com/BioInfoTools/BBMap). Reads mapped to assemblies to calculate relative abundance data were trimmed as above, but not normalised or error corrected. FAST5 reads from VirION libraries were basecalled with Albacore and processed with Porechop to remove adapters from both ends and to split reads containing middle adapters. Processed reads were filtered by NanoFilt to keep sequences with a q-score >=10. To ensure removal of adapters, the first 50 bases were then removed from the reads. Reads less than 1 kbp in length were also discarded.

Evaluating metagenomic assembly of long reads in Canu in mock community and environmental virome data

Overlap-layout consensus assembly of VirION reads was performed using Canu (Koren et al., 2017) with the following parameters: “genomeSize=180m minReadLength=1000 contigFilter="2 1000 1.0 1.0 2" corOutCoverage=999 correctedErrorRate=0.040 -nanopore-raw”. *genomeSize* parameter selection was optimised by testing values from 9-180 Mbp data. Above a value of 45 Mbp, all assemblies were nearly identical, so the largest value was chosen for subsequent assembly. All assembly comparisons were performed using and metaQUAST (Mikheenko, Saveliev & Gurevich, 2016).

Evaluating chimerism and inclusion of long-reads in recovery of genomes from a mock viral community

Short, hybrid and long-read assemblies of VirION reads from the mock viral community (Table S1) were compared using metaQUAST (Mikheenko, Saveliev & Gurevich, 2016). Quantification of error rates in short and long read sequences were calculated with samtools (Li et al., 2009). Chimeric raw reads were identified as those that had two alignments > 100 bp that did not represent alignments to both the start and end of the genome (to avoid counting reads that mapped across an *in-silico* breakage of a circular genome into a linear representation). Mock community assemblies were evaluated for chimeric assemblies using MUMmer (Delcher, Salzberg & Phillippy, 2003) against member genomes and identifying those that aligned to more than one member.

Evaluating impact of multi-kilobase LASL PCR amplification on read relative abundance

Relative abundance of mock viral community members in short-read and VirION datasets was evaluated by mapping high quality short reads and long reads against the genomes of mock viral community members using bowtie2 (Langmead & Salzberg, 2012) and minimap2 (https://github.com/lh3/minimap2), respectively, followed by calculation of relative abundance using samtools (Li et al., 2009).

Evaluating capture of more microdiverse viral genomes using long-read assembly

For short, hybrid and long-read polished assemblies, nucleotide diversity was calculated as follows: High quality short reads from the Western English Channel were mapped back to viral contigs and reads mapping at <95% identity to any viral contig were removed to evaluate nucleotide diversity within established parameters for viral populations (Brum *et al.,* 2015). Contigs with >10-fold coverage across 70% of their whole genome were retained for robust calculation of the ratio of non-synonymous to synonymous polymorphism rates (Schloissnig *et al*., 2013; Brum *et al.,* 2015). Single nucleotide polymorphisms (SNPs) were identified using mpileup and BCFtools (https://samtools.github.io/bcftools/bcftools.html) and those with a quality score >=30, represented by at least 4 reads and comprising >1% of the base pair coverage for that position were considered true SNPs. SNP frequencies across all genomes were rarefied by subsampling to 10[illegible] coverage proportionate to the frequency of different SNPs per site while maintaining SNPs linkages. Parameters were chosen for conservative estimates, in accordance with previously published work (Schloissnig *et al*., 2013). Observed nucleotide diversity ([illegible]) (Nei & Li, 1979) was estimated both per contig (median across the length of the contig) and at a per-base level.

Evaluating the recovery of genomic islands by inclusion of long-reads in viral metagenomic assembly

We identified genomic islands in viral contigs from short-read only assembly, hybrid assembly and polished long-read assembly of VirION reads, as described previously (Mizuno, Ghai & Rodriguez-Valera, 2014), with parameter optimisation to account for increased error in long reads Short read data from the Western English Channel was mapped back against the viral contigs > 10kb using bowtie2 (Langmead & Salzberg, 2012) and samtools (Li et al., 2009). BAM files were filtered using BamM (http://github.com/ecogenomics/BamM) to remove reads mapping at nucleotide identities ranging from 92-98%, to assess any impact of increased sequencing error in long-read assemblies. Contigs with a median per-base coverage of < 5 or those with a Reads per kb of genome per Gbp of reads mapped (RPKG) of < 1 were identified with BamM and removed from analysis. Genomic islands were defined as regions where the median coverage of a 500 bp sliding window was < 20% of the median coverage of the contig (Mizuno, Ghai & Rodriguez-Valera, 2014). Putative genomic islands were excluded if they were within 500 bp of the end of a contig. If two genomic islands were found within 500 bp of each other, they were combined into a single genomic island. Lengths of genomic islands and density of genomic islands per contig were calculated for each assembly type. Effect size of different assembly types on genomic island length and density and associated 95% confidence intervals (CI) were calculated from bootstrapped medians (Cumming, 2014).

Evaluating gene content within viral genomic islands using long reads

As each long read is amplified from a single template strand, it is possible to investigate how conserved the protein content is within genomic islands across viral populations by identifying reads that have different protein content, but which span the same genomic island. First, individual long reads were error corrected with short reads as described previously. Corrected reads with a minimum of 5-fold average coverage for consensus basecalling were selected and mapped back against viral contigs > 10 kbp using minimap2 (https://github.com/lh3/minimap2). Genomic islands with at least 10 VirION reads spanning the full length of the island were selected for further study (137 genomic islands on 84 viral contigs). Median length of predicted proteins on these reads was 75 amino acids (74-76 aa, 95% CI) showing error correction had not been sufficient to correct for the enriched presence of stop codons resulting from indel errors. Therefore, an alternative approach to gene calling was developed. VirION reads

spanning genomic islands were trimmed at the 5' and 3' end to leave only the read fragment mapping within the genomic island, and these fragments were used as a query in a BLASTx search against the NR database using diamond (Buchfink, Xie & Huson, 2015), with the following settings:

'-k 500 –more-sensitive –frameshift 15 –subject-cover 20 –evalue 1e-5'

Each read fragment had the potential to encode several proteins, along its length, with each putative gene aligning to several similar proteins in the NR database. This information was used to estimate the start and stop loci of each putative gene using an unsupervised learning approach. Identification of the start and stop loci for each putative gene was estimated by hierarchically clustering start and stop loci for each match to an NR protein using Euclidian distance and single linkage. A threshold of 200 bp was used to discriminate between clusters following evaluation of a subset of 100 randomly selected reads at 50, 100, 200 and 500 bp). Below is an example of this clustering performed on read 01360d0f-19c8-4725-9c0f-e28b7b0db8a0_Basecall_2D_2d that mapped to contig P_tig00000038_pilon across its genomic island at 63060-64955 bp. In this example, clustering identified three separate gene products. Minimum start and maximum end loci of each cluster were used to extract putative gene encoding fragments from the VirION reads.

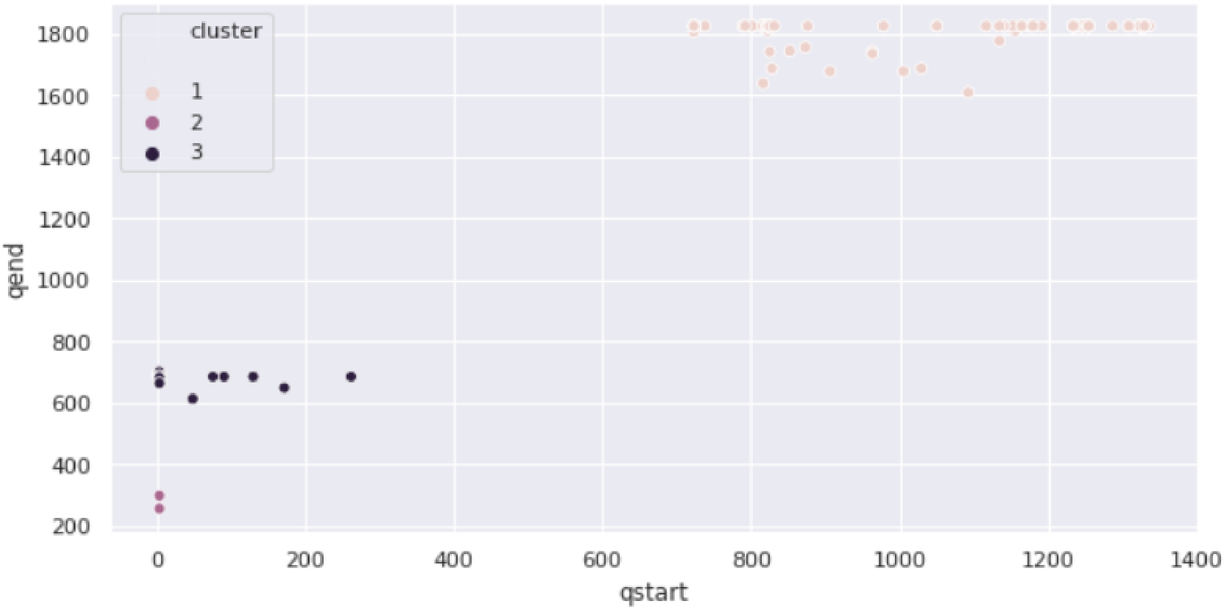

Extracted gene fragments were then used as a query against NR using diamond BLASTX as before, but this time returning only the top hit and requiring at least 20 % of the query matched the subject. In total, 6,445 fragments were extracted from 3,072 reads, of which 4,599 returned a hit to a subject within the NR database. Of these, 3,888 (85%) were assigned to proteins whose title included the words 'hypothetical', 'DUF' (domain of unknown function), 'unknown' or 'uncharacterized'. The remaining 711 hits were then used to calculate the total number of unique annotated reads spanning each GI, the predicted functions within each GI and the number of different taxa identified within each GI. To evaluate whether different VirION reads spanning the same GI encoded different functions, two further criteria were evaluated. Firstly, all proteins hits associated with a genomic island were compared in an all-vs-all BLASTP (expect threshold: 10; word size: 6; matrix: BLOSUM62; gap costs: existence 11; Extension: 1). Different function was

assumed if proteins had an alignment score <40. Secondly, start coordinates of different functional genes within a GI were compared to evaluate if one function was adjacent to another in the same genome, rather than being encoded in the same locus, but in different viral genomes. Different functional genes were conservatively assumed to be adjacent if they had an overlap of <100 bp to account for inaccurate identification of the start and end loci on error-prone reads. Results are available in Table S5.

References:

Buchfink B, Xie C, Huson DH. 2015. Fast and sensitive protein alignment using DIAMOND. *Nature Methods* 12:59–60.

Bolger AM., Lohse M., Usadel B. 2014. Trimmomatic: a flexible trimmer for Illumina sequence data. *Bioinformatics* 30:2114–2120.

Brum, J. R., Ignacio-Espinoza, J. C., Roux, S., Doulcier, G., Acinas, S. G., Alberti, A., … Sullivan, M. B. (2015). Ocean plankton. Patterns and ecological drivers of ocean viral communities. *Science*, 348(6237), 1261498.

Darling ACE, Mau B, Blattner FR, Perna NT. 2004. Mauve: multiple alignment of conserved genomic sequence with rearrangements. Genome Research 14:1394–1403.

Kearse M, Moir R, Wilson A, Stones-Havas S, Cheung M, Sturrock S, Buxton S, Cooper A, Markowitz S, Duran C, Thierer T, Ashton B, Meintjes P, Drummond A. 2012. Geneious basic: an integrated and extendable desktop software platform for the organization and analysis of sequence data. Bioinformatics 28:1647–1649.

Li H, Handsaker B, Wysoker A, Fennell T, Ruan J, Homer N, Marth G, Abecasis G, Durbin R, 1000 Genome Project Data Processing Subgroup. 2009. The sequence alignment/map format and SAMtools. Bioinformatics 25:2078–2079.

Mikheenko A, Saveliev V, Gurevich A. 2016. MetaQUAST: evaluation of metagenome assemblies. Bioinformatics 32:1088–1090.

Schloissnig, S., Arumugam, M., Sunagawa, S., Mitreva, M., Tap, J., Zhu, A., Waller A, Mende DR, Kultima JR, Martin J, Kota K, Sunyaev SR, Weinstock GM, Bork, P. (2013). Genomic variation landscape of the human gut microbiome. *Nature*, 493(7430), 45–50.
