# Supplementary material for: Long-read viral metagenomics captures abundant and microdiverse viral populations and their niche-defining genomic islands
Source: PeerJ. 2019 Apr 25;7:e6800. doi: 10.7717/peerj.6800 (PMC6487183; doi:10.7717/peerj.6800)
Supplement: Table S1 — Genomic characteristics of the six phages chosen for the mock viral community to develop and evaluate VirION protocols. [file peerj-07-6800-s002.docx]

**Supplementary Table 1.** **Mock viral community member characteristics**

| Phage | Taxonomy | GC (%) | Genome Size (kbp) |
| --- | --- | --- | --- |
| *Pseudoalteromonas* phage HM1 | Myoviridae | 35.7 | 129.4 |
| *Cellulophaga* phage 38:1 | Podoviridae | 38.1 | 72.5 |
| *Cellulophaga* phage 38:2 | Myoviridae | 33.5 | 54.0 |
| *Pseudoalteromonas* phage HP1 | Podoviridae | 44.7 | 45.0 |
| *Cellulophaga* phage 18:1 | Siphoviridae | 36.5 | 39.2 |
| *Pseudoalteromonas* phage HS2 | Siphoviridae | 40.2 | 38.2 |
