# Supplementary material for: Long-read viral metagenomics captures abundant and microdiverse viral populations and their niche-defining genomic islands
Source: PeerJ. 2019 Apr 25;7:e6800. doi: 10.7717/peerj.6800 (PMC6487183; doi:10.7717/peerj.6800)
Supplement: Table S2 — For comparison important viral metagenomic studies (see references) and viruses from ‘RefSeq’. Prior to quantification of global relative abundances and (shared-protein) clustering, phage genomes were re-analysed using VirSorter to ensure uniformity of gene-calling, resulting in above classifications. Note: VirSorter Categories as follows: 1 and 4: “most confident” predictions (viral and lysogen, respectively); 2 and 5: “likely” predictions (viral and lysogen, respectively). [file peerj-07-6800-s003.docx]

**Supplementary Table 2. The numbers of phage genomes from identified in this study using short, hybrid and error-corrected long read assembly of VirION reads, as identified by VirSorter** [(Roux et al., 2015)](https://paperpile.com/c/ssJt3i/qxHI)**. For comparison important viral metagenomic studies (see references) and viruses from ‘RefSeq’**

| VirSorter Category | WEC short | WEC hybrid | WEC long | GOV [(Roux et al., 2016a)](https://paperpile.com/c/ssJt3i/97zA) | Luo 2017 [(Luo et al., 2017)](https://paperpile.com/c/ssJt3i/7TZi) | vSAG [(Martinez-Hernandez et al., 2017)](https://paperpile.com/c/ssJt3i/1Ay3) | RefSeq |
| --- | --- | --- | --- | --- | --- | --- | --- |
| 1 | 158 | 213 | 56 | 2024 | 112 | 3 | 473 |
| 2 | 715 | 1173 | 305 | 11871 | 214 | 34 | 466 |
| 4 | 0 | 1 | 0 | 3 | 0 | 0 | 0 |
| 5 | 6 | 13 | 5 | 37 | 0 | 0 | 9 |
| Total | **879** | **1400** | **366** | **13935** | **326** | **37** | **948** |

Prior to quantification of global relative abundances and (shared-protein) clustering, phage genomes were re-analysed using VirSorter to ensure uniformity of gene-calling, resulting in above classifications. Note: VirSorter Categories as follows: 1 and 4: “most confident” predictions (viral and lysogen, respectively); 2 and 5: “likely” predictions (viral and lysogen, respectively).
