# Supplementary material for: Long-read viral metagenomics captures abundant and microdiverse viral populations and their niche-defining genomic islands
Source: PeerJ. 2019 Apr 25;7:e6800. doi: 10.7717/peerj.6800 (PMC6487183; doi:10.7717/peerj.6800)
Supplement: Table S3 — Student t-test results to identify significant differences between the number of circular viral contigs (as identified by VirSorter (Roux et al., 2015) from short read only vs. hybrid assemblies with VirION reads using metaSPAdes assemblies from triplicate random subsamples of short reads across different levels of sequencing depth. Significant differences are highlighted in bold. [file peerj-07-6800-s004.docx]

**Supplementary Table 3: Student t-test results to identify significant differences between the number of circular viral contigs (as identified by VirSorter** [(Roux et al., 2015)](https://paperpile.com/c/ssJt3i/qxHI)**) from short read only vs. hybrid assemblies with VirION reads using metaSPAdes assemblies from triplicate random subsamples of short reads across different levels of sequencing depth.** Significant differences are highlighted in bold.

| **Sequencing depth (Gbp)** | ***p*-value** | **Mean count of circular viral contigs (short-read only)** | **Mean count of circular viral contigs (hybrid)** | **Fold-difference** |
| --- | --- | --- | --- | --- |
| 3.08 | 0.26 | 8.00 | 13.7 | 1.71 |
| 6.17 | 0.08 | 11.7 | 22.7 | 1.94 |
| 9.25 | 0.05 | 18.3 | 31.00 | 1.70 |
| **12.34** | **0.00** | **20.7** | **33.3** | **1.61** |
| **15.42** | **0.04** | **18.7** | **36.7** | **1.96** |
| **18.50** | **0.01** | **23.3** | **36.0** | **1.54** |
| **21.59** | **0.01** | **25.3** | **39.7** | **1.56** |
