# Supplementary figures and images for: Long-read viral metagenomics captures abundant and microdiverse viral populations and their niche-defining genomic islands
Source: PeerJ. 2019 Apr 25;7:e6800. doi: 10.7717/peerj.6800 (PMC6487183; doi:10.7717/peerj.6800)

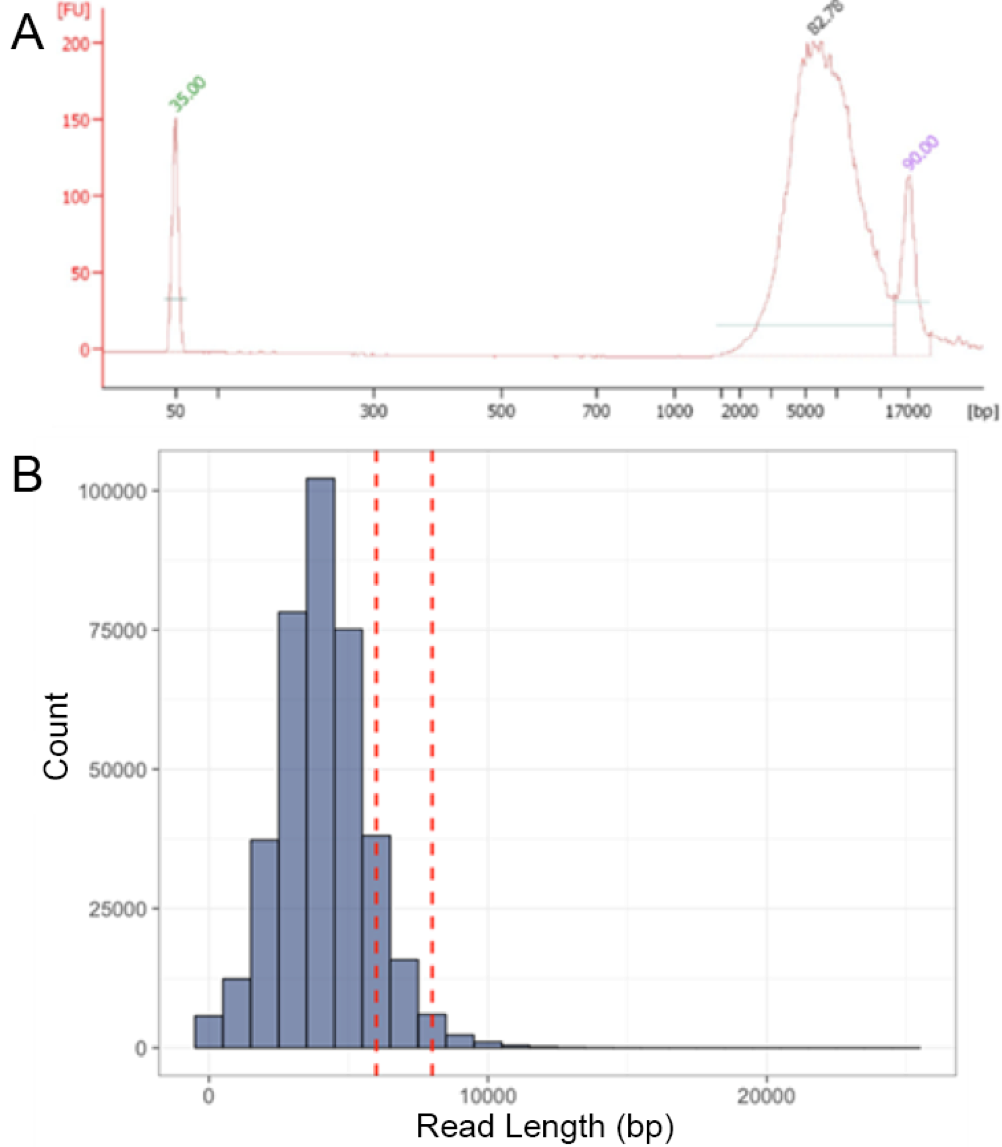

Supplement: Figure S1 — (A) Bioanalyzer (Agilent) electropherogram showing the fragment length distribution of linker-amplified mock viral community DNA produced from 20 ng template DNA sheared to ∼8kbp. Amplicon length peaked at ∼5.4 Kbp, demonstrating PCR preference for amplification of shorter DNA fragments; (B) Read length distribution of VirION mock viral community amplicons (as shown in ‘A’; red dashed lines indicate approximate length of sheared template DNA); mean average read length was ∼4 kbp, likely due to preferential sequencing of shorter DNA fragments. [file peerj-07-6800-s007.png]

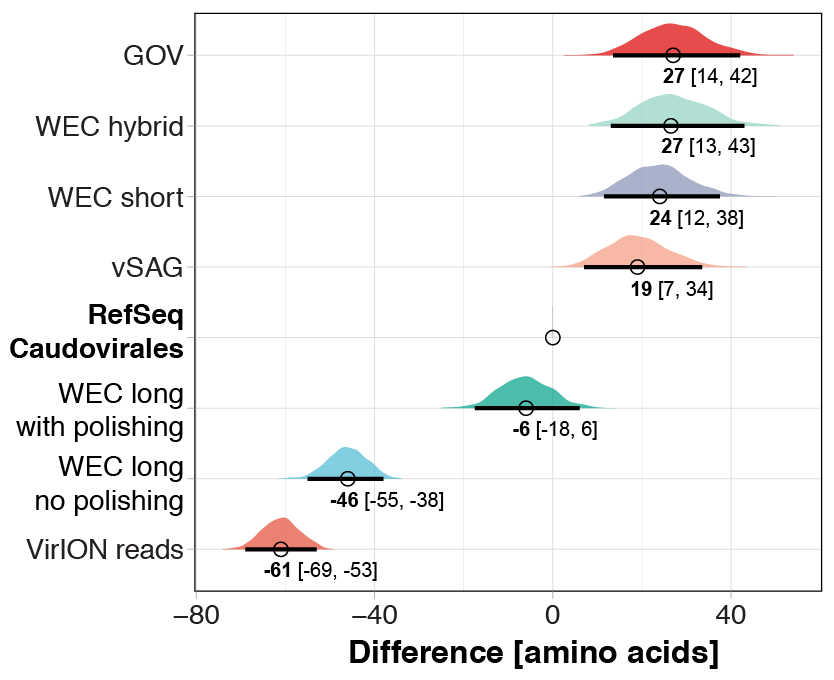

Supplement: Figure S3 — Median predicted protein length of 1,000 randomly selected proteins were calculated and compared to a similar treatment of proteins from a RefSeq v.8.4 Caudovirales database to measure effect size. This process was bootstrapped 1,000 times to provide 95% confidence intervals. The distributions on the graph represent distributions of differences in medians (Cumming, 2014) . The median effect size (bold number) and the 95% CI boundaries (black line under each distribution, and numbers in brackets) are shown. [file peerj-07-6800-s009.png]

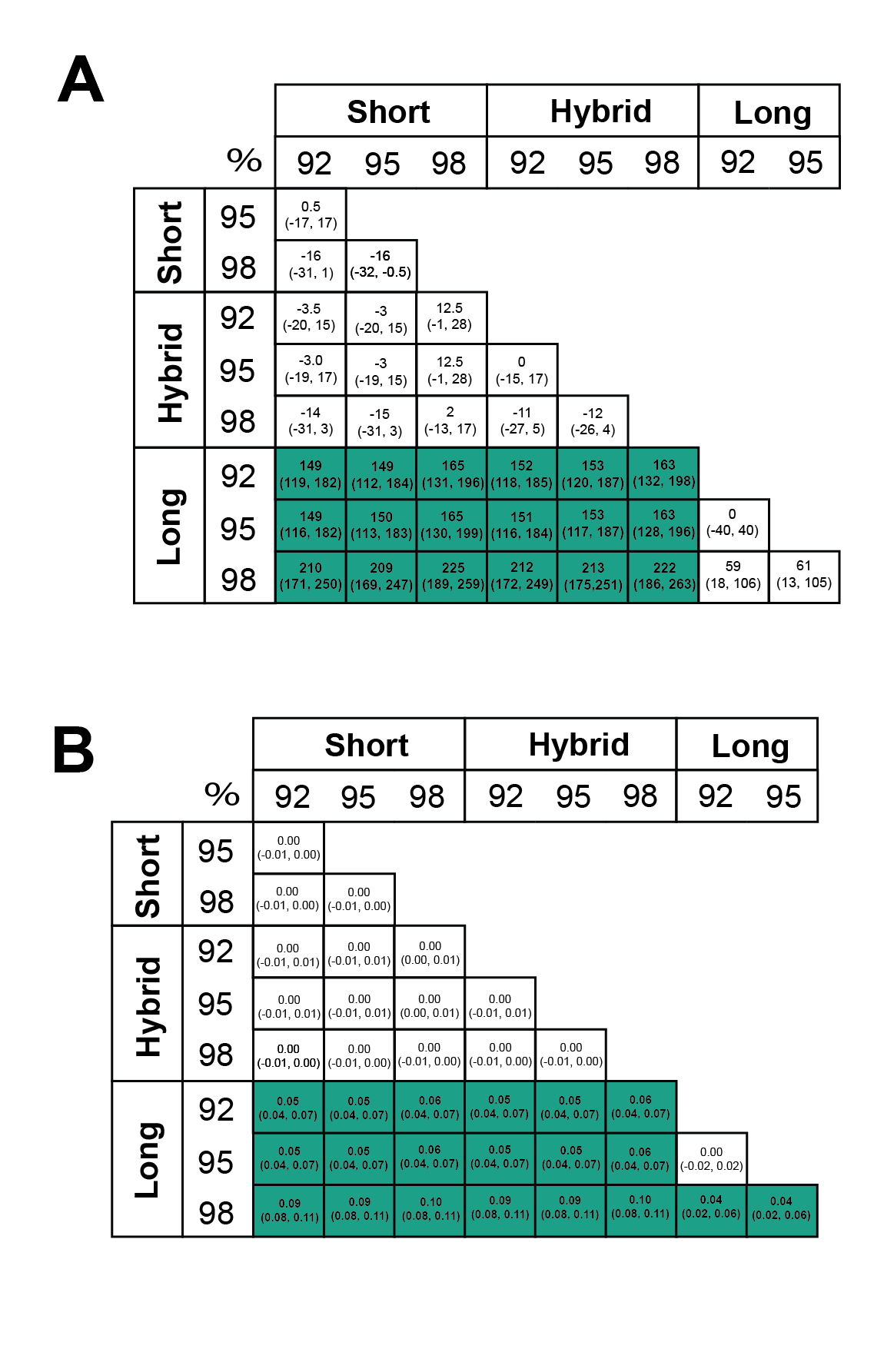

Supplement: Figure S4 — Effect size and bootstrapped median 95% CI intervals for impact of different assembly types on (A) genomic island length and (B) genomic island density (kbp of genomic island per kbp of genome). Values in boxes represent the median difference between 1,000 bootstrapped medians (95% CI). Green boxes represent significant ( p < 0.05) differences calculated with a Wilcoxon Rank Sum test. [file peerj-07-6800-s010.png]

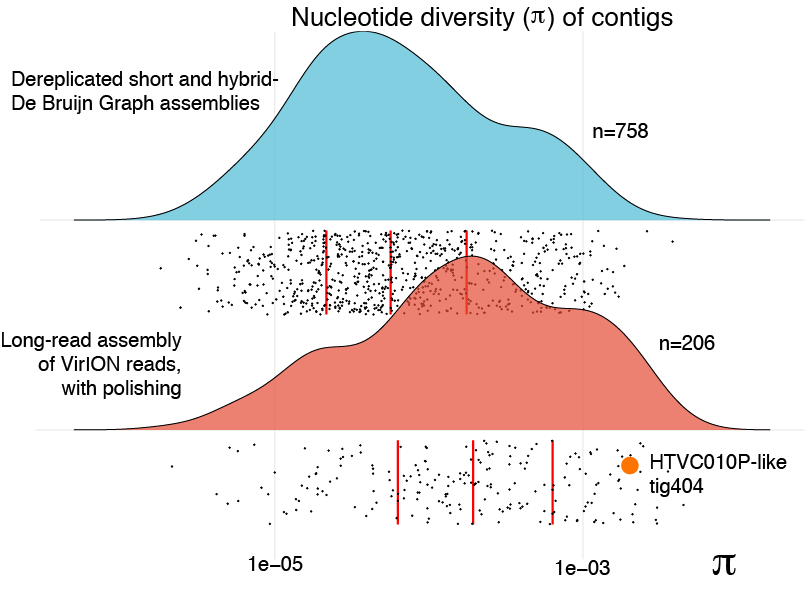

Supplement: Figure S5 — The data point for long-read assembled contig tig404 (described in the main text) is highlighted; this virus belongs in the same viral cluster as pelagiphage HTVC010P, an abundant phage that fails to assemble in metagenomic datasets, potentially due to high microdiversity. [file peerj-07-6800-s011.png]

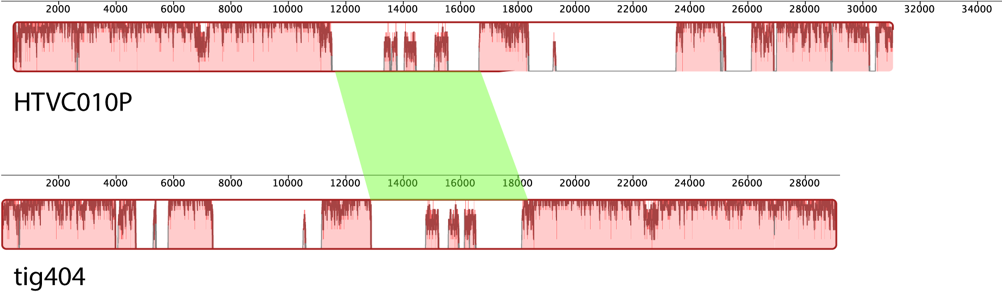

Supplement: Figure S6 — Genomes were 89% identical at nucleotide in shared regions and both shared a conserved genomic island (green) bounded by structural proteins. Genome alignments were produced by Mauve (Darling et al., 2004) within the Geneious software (Kearse et al., 2012). [file peerj-07-6800-s012.png]

# Top 50 most abundant viral contigs in L4 sample

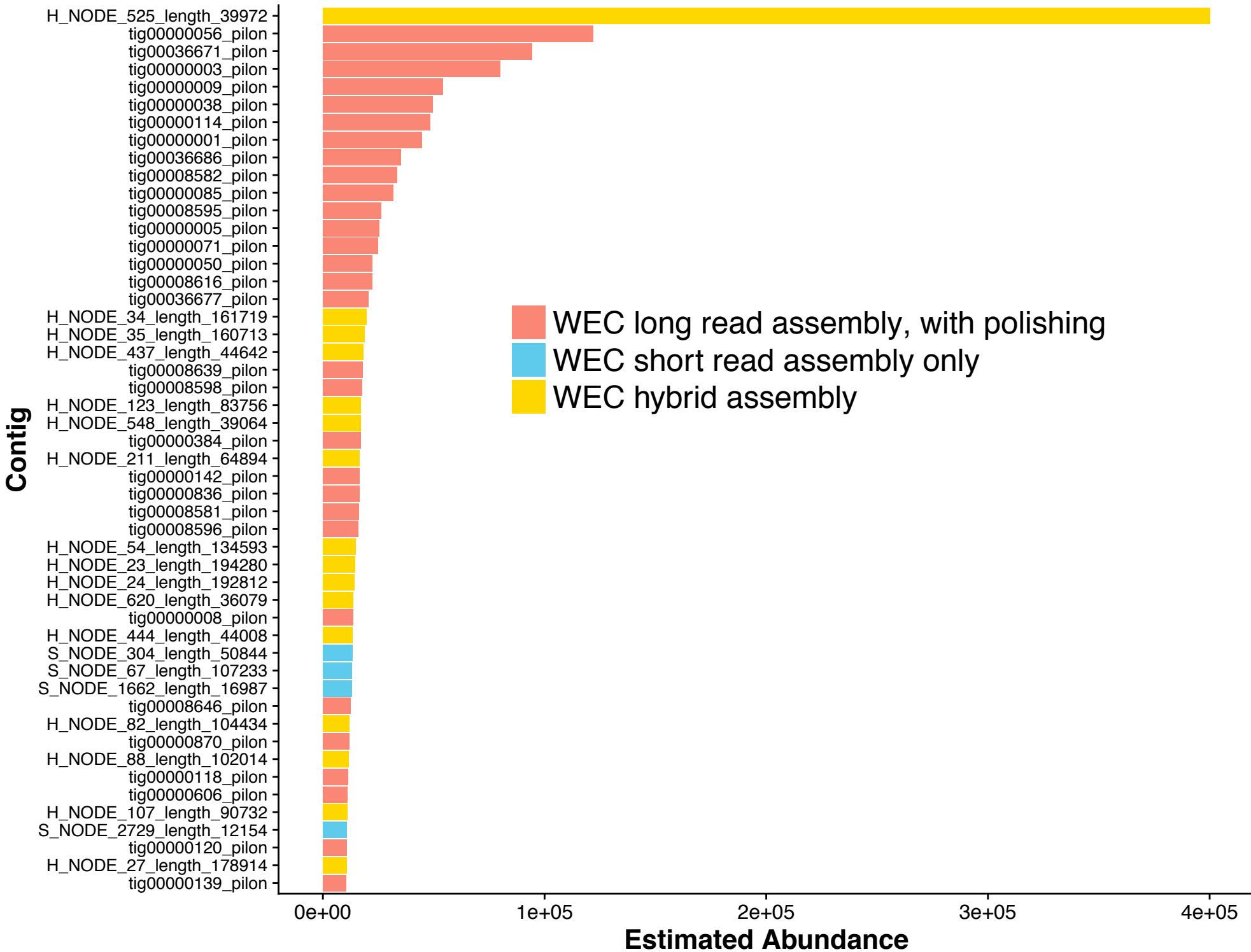

Supplement: Figure S7 — Estimated relative abundances (number of recruited reads from the short-read dataset) of the Western English Channel viral contigs were calculated by competitive recruitment of short reads back to viral contigs derived from the VirION bioinformatics pipeline using FastViromeExplorer (Tithi et al., 2018). 60% of the top 50 most abundant viruses are detected only in the error-corrected overlap layout consensus assemblies. [file peerj-07-6800-s013.pdf]

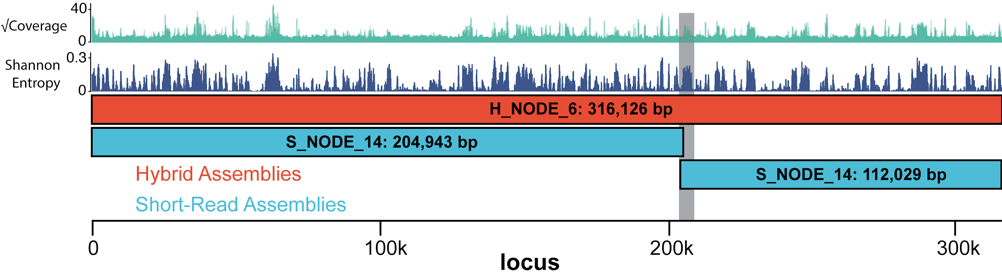

Supplement: Figure S8 — H_NODE_6 was the longest recovered virus captured by scaffolding of a De Bruijn Graph assembly using VirION reads (red). Alignment of short read only contigs (blue) against the complete genome show the full length is only captured by the scaffolding approach, whereas the short-read approach results in a breakage at ∼205 kbp (grey box). Coverage and Shannon Entropy are both shown as median values of a 200 bp sliding window, with 100 bp overlap. [file peerj-07-6800-s014.png]
