# Supplementary material for: Long-read viral metagenomics captures abundant and microdiverse viral populations and their niche-defining genomic islands
Source: PeerJ. 2019 Apr 25;7:e6800. doi: 10.7717/peerj.6800 (PMC6487183; doi:10.7717/peerj.6800)
Supplement: Figure S2 — Impact of using short read sequencing to error correct overlap layout consensus-derived contigs with Pilon shows that approximate limits of the number of insertions and deletions that can be fixed is reached at ∼9 Gbp of short read data (median coverage of ∼70). Analysis was performed against the full contig set from Overlap layout consensus assembly of VirION reads from the Western English Channel ( n = 1,500). [file peerj-07-6800-s008.pdf]

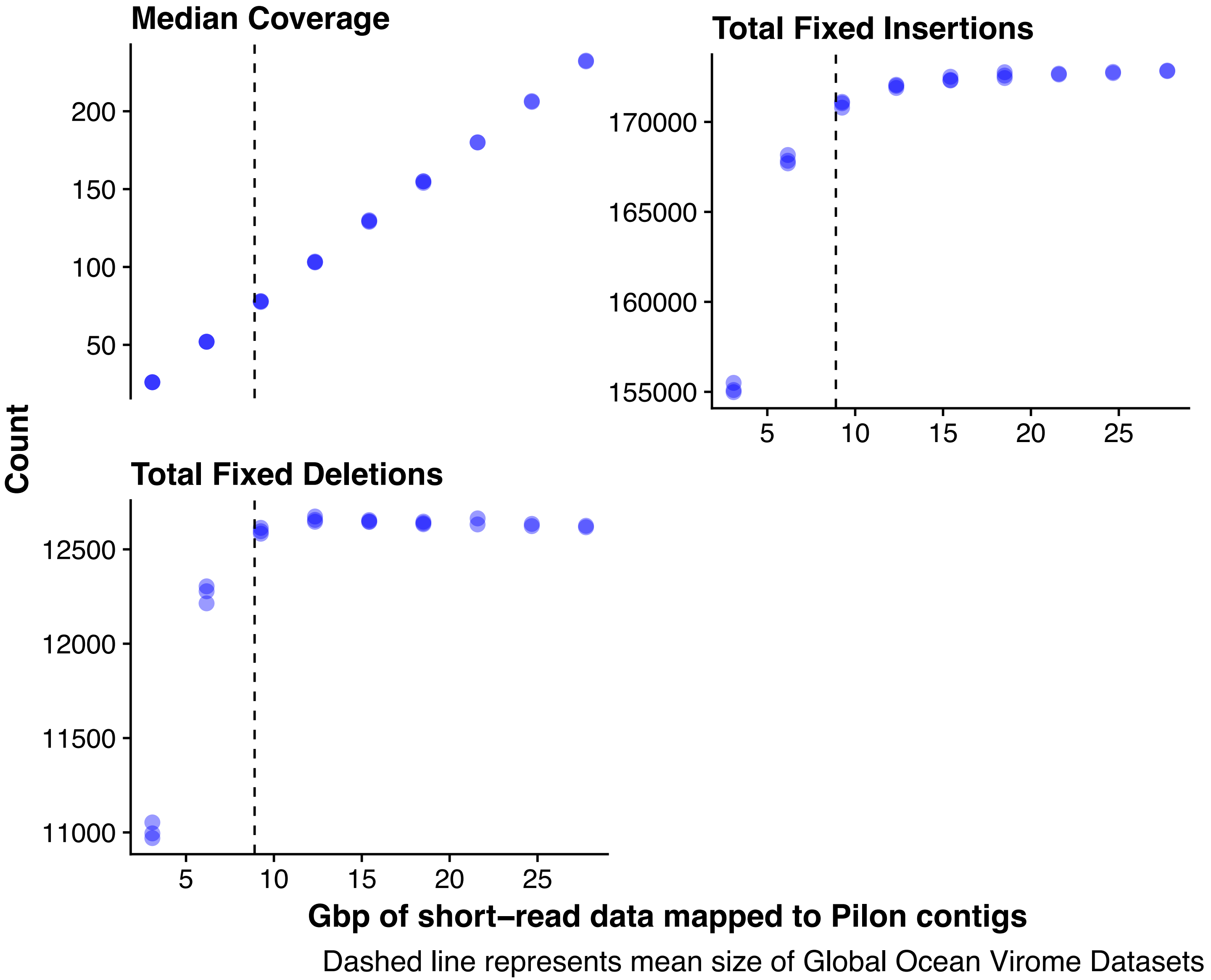

Median Coverage
200
150
100
50
Total Fixed Insertions
170000
165000
160000
155000
5
10
15
20
25
Count
Total Fixed Deletions
12500
12000
11500
11000
5
10
15
20
25
Gbp of short−read data mapped to Pilon contigs
Dashed line represents mean size of Global Ocean Virome Datasets
